# Prevalence of polycystic ovary syndrome among college students in kashmir (J&K) india: a cross-sectional study

**DOI:** 10.3389/fendo.2026.1912986

**Published:** 2026-08-26

**Authors:** Mohd Anis Ganaie, Mir Tanveerul Hassan, Amrit Sudershan, Ruheena Jawaid Raina, Maria Shah, Misbah Ul Fidda, Agar Chander Pushap, Saima Gaffar, Sang Jun Lee

**Affiliations:** 1 Department of Zoology, Sri Pratap College Cluster University Srinagar, J&K, Srinagar, India; 2 Jeonbuk RICE Intelligent Innovation Research Centre, Jeonbuk National University, Jeonju, Republic of Korea; 3 Department of Human Genetics, Sri Pratap College, Cluster University Srinagar, J&K, Srinagar, India; 4 Department of Education, Dakshina Bharat Hindi Prachar Sabha, Chennai, Tamil Nadu, India; 5 Department of Electronics and Information Engineering, Jeonbuk National University, Jeonju, Republic of Korea

**Keywords:** endocrine disorder, hyperandrogenism, polycystic ovary syndrome, reproductive health, Rotterdam criteria

## Abstract

**Background:**

Polycystic Ovary Syndrome (PCOS) is a common endocrine disorder and a major cause of infertility among reproductive-age women. Despite its high prevalence, awareness and early diagnosis remain inadequate, particularly among young women, and epidemiological data on college-aged women in the Indian subcontinent, including Kashmir, are limited.

**Aim:**

This study aimed to estimate the prevalence of self-reported and symptom-screened PCOS and evaluate awareness among female university students in the Kashmir division. By identifying symptom patterns and knowledge gaps, it provides evidence to support early detection and targeted educational interventions.

**Method:**

A cross-sectional study was conducted between July and December 2024 among female students from randomly selected colleges in the Kashmir region. A total of 1,217 participants were enrolled using convenience sampling. Data were collected using a structured, pre-validated questionnaire assessing demographics, PCOS awareness, and symptoms. Participants with a self-reported previous diagnosis of PCOS were excluded from symptom screening. The psychometric properties of the 20-item PCOS knowledge questionnaire were evaluated using the Kuder–Richardson Formula 20 (KR-20) and a two-parameter Logistic Item Response Theory (IRT) model.

**Result:**

The mean age of participants was 21.86 ± 2.24 years. Among participants without a previous diagnosis of PCOS, 33.64% were identified as being at risk of PCOS and 1.28% had symptom profiles highly suggestive of PCOS. Additionally, 29.90% of participants reported a previous diagnosis of PCOS. Awareness of the broader health implications of PCOS remained inadequate. Although gynecological symptoms were generally well recognized, knowledge of metabolic consequences and long-term complications was limited. Less than half of the participants were aware of the association between PCOS and insulin resistance (44.53%), type 2 diabetes (45.02%), and cardiovascular disease (33.00%).

**Discussion & conclusion:**

The findings suggest a substantial burden of previously undiagnosed, symptom-screened PCOS and inadequate awareness of its long-term health implications. Targeted health education, early screening, and appropriate clinical evaluation are needed to improve PCOS awareness and management.

## Graphical Abstract

**Figure:**
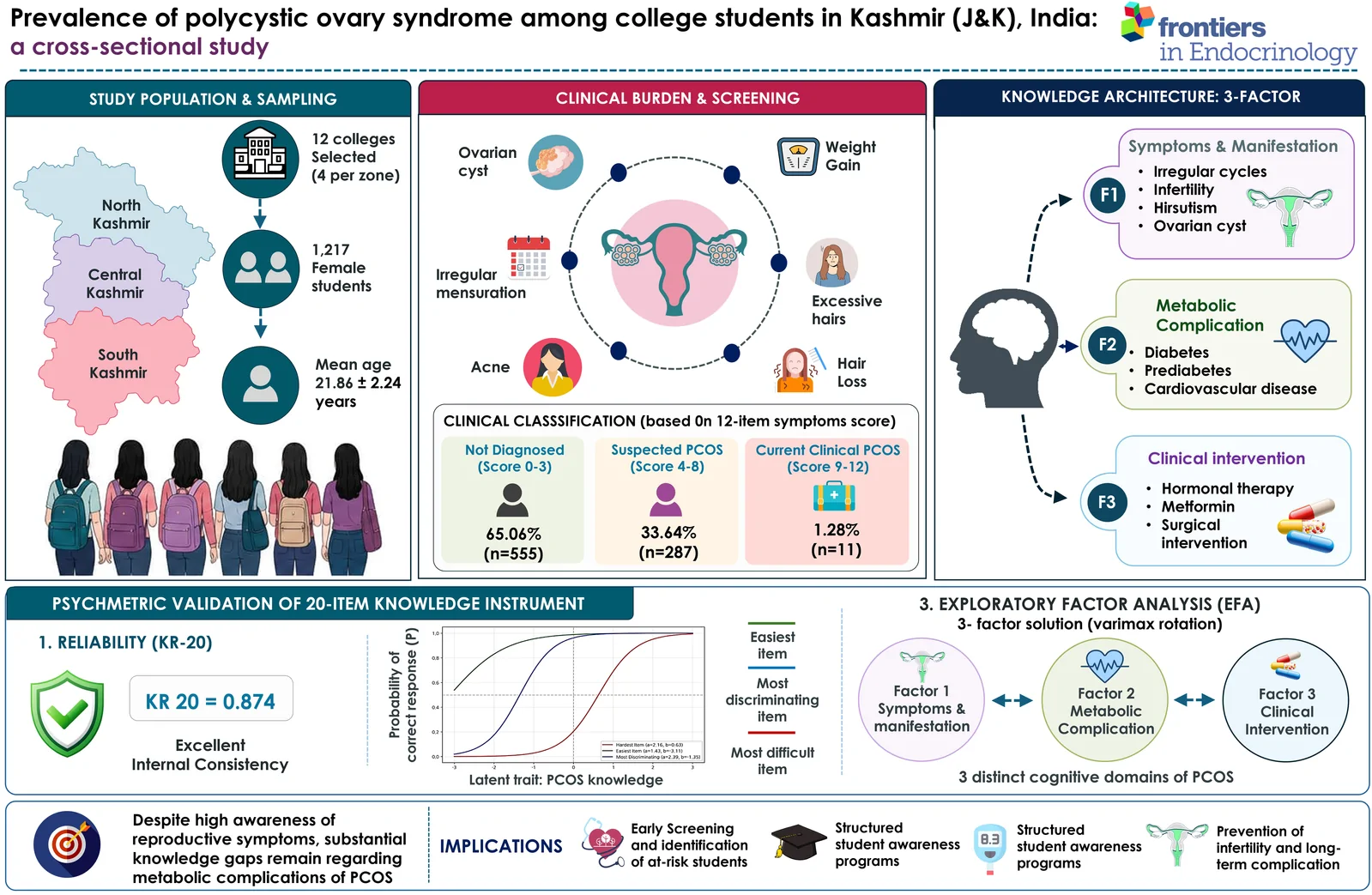

## 1 Introduction

Polycystic Ovary Syndrome (PCOS), recently named as Polyendocrine Metabolic Ovarian Syndrome (PMOS), is a common and lifelong hormonal disorder that affects women of reproductive age and is one of the major causes of infertility worldwide, contributing to nearly 70% of ovulation-related infertility cases (1–3). PCOS primarily disrupts ovarian function and hormonal balance, resulting in menstrual irregularities, increased levels of male hormones (hyperandrogenism), and the formation of multiple ovarian cysts detectable by ultrasonography (4–6). Although the precise etiology of PCOS remains unclear, its development is believed to involve genetic, hormonal, metabolic and environmental factors (7). A family history of the disorder further increases susceptibility, highlighting the significant role of genetic predisposition in its pathogenesis (8). PCOS is associated with significant reproductive complications, including infertility, adverse pregnancy outcomes and an increased risk of endometrial carcinoma, primarily due to chronic anovulation and abnormal endometrial proliferation (9). From a metabolic perspective, PCOS is closely associated with obesity, metabolic syndrome, type 2 diabetes mellitus (T2DM), dyslipidemia, hypertension, and cardiovascular diseases. Studies indicate that many women with PCOS develop impaired glucose tolerance by early adulthood and have an increased risk of progressing to diabetes and hypertension. However, evidence suggests that both metabolic and reproductive complications can be improved through healthy lifestyle modifications (10). Due to its high prevalence and long-term health consequences, PCOS is considered a major public health concern requiring early screening and multidisciplinary management. Diagnosis of PCOS is based on clinical symptoms, hormonal evaluation, and ultrasound findings, as no single test can confirm the condition. The widely accepted Rotterdam criteria diagnose PCOS when at least two of the following features are present: irregular or absent ovulation, signs of excess male hormones, and polycystic ovaries on ultrasound. These criteria reflect the complex and diverse nature of the disorder, first described by Irving F. Stein Sr. and Michael L. Leventhal in 1935 (11).

Given its multifactorial nature and long-term health consequences, understanding the prevalence of PCOS in different populations is essential for improving disease management and healthcare planning. According to the World Health Organization, PCOS affects 8%–13% of women globally, with nearly 70% of cases remaining undiagnosed. In India, prevalence ranges from 2% to 35%, with particularly high rates reported in Kashmir (29%–35%) (12). However, data on college-aged women, an age group where symptoms often first appear, remain limited. Therefore, this cross-sectional study aims to assess the prevalence and awareness of PCOS among female university students. Early diagnosis and better awareness are essential for preventing complications such as infertility and metabolic disorders.

## 2 Methods

### 2.1 Ethical permission & consent to participate

This study was conducted in accordance with established ethical guidelines. The research protocol was reviewed and approved by the Institutional Ethics Committee (IEC) of Sri Pratap College, Cluster University Srinagar, Kashmir, India (IEC No. SPC:1323; dated 04-05-2024). All participants were informed about the objectives and purpose of the study through an online information sheet, and digital informed consent was obtained prior to participation. Participation was entirely voluntary, and the anonymity and confidentiality of participant information were strictly maintained throughout the study.

### 2.2 Study design, sampling method, and sample size determination

A cross-sectional study was conducted over a six-month period from July to December 2024 across colleges in the Kashmir division, after obtaining ethical approval. A multistage sampling approach was adopted. Initially, a comprehensive list of colleges was prepared using Microsoft Excel, and colleges from rural and urban areas were randomly selected using the RAND function to ensure diverse regional representation. Since Kashmir is divided into three geographical zones (North Kashmir, South Kashmir, and Central Kashmir), four colleges from each zone were selected. Subsequently, participants were recruited using convenience sampling due to logistical and resource constraints. Female college students aged 18–26 years who provided informed consent were included in the study. Male respondents, incomplete responses, and participants unwilling to participate were excluded. Data collection was carried out using a structured questionnaire. Although convenience sampling may introduce selection bias, it was considered appropriate for achieving timely recruitment across a large geographic area. It is important to note that participant recruitment was conducted through a voluntarily accessible via e-Form distributed within the selected colleges using convenience sampling. Consequently, the total number of students invited to participate and the overall response rate were not prospectively recorded.

**Figure 1:**
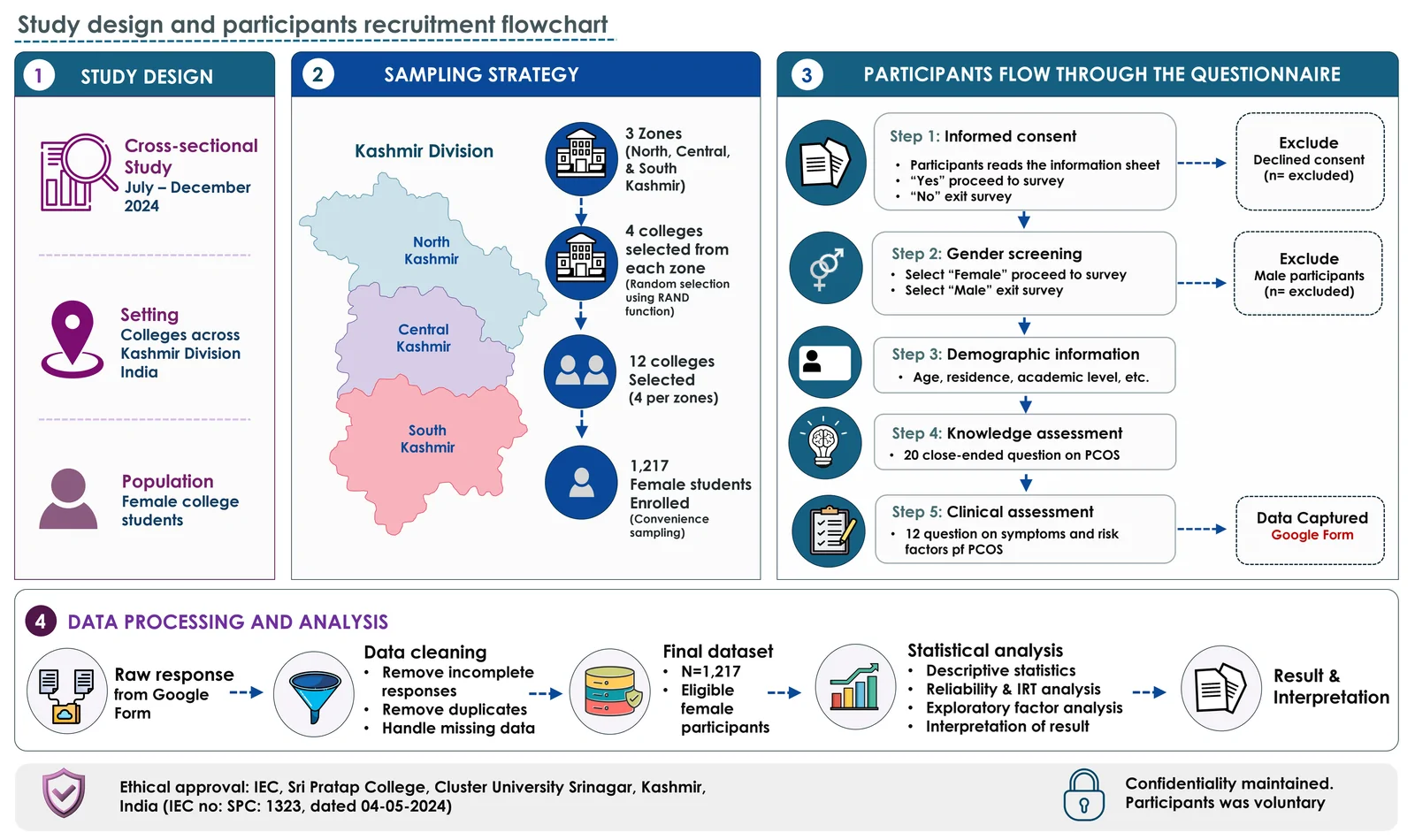
Schematic overview of the study methodology and participant selection process.

The overall workflow of the study, including the sampling strategy, clinical burden assessment, questionnaire validation, and factor structure identification is summarized in **Figure 1**.

The minimum sample size was calculated using the standard prevalence formula 1:

(1) $$n\;=\;\frac{\left(Z^{2}\;\times \;P\;\times \;(1\;-\;P)\right)}{d^{2}}$$

where *Z* = 1.96 at a 95% confidence level, *P* = 0.50, and *d* = 0.05. Based on these parameters, the minimum required sample size was 384 participants. However, to improve the robustness and representativeness of the findings, data were collected from 1,217 participants across the selected colleges.

### 2.3 Questionnaire design and administration

Data were collected using a closed-ended, structured questionnaire adapted from previously published studies (13–15). The questionnaire consisted of three sections. The first section collected demographic information, the second included 20 questions assessing participants’ knowledge regarding PCOS, and the third comprised 12 symptom-based questions related to the clinical features of PCOS. The questionnaire was originally developed and administered in English, as the participants possessed sufficient language proficiency to understand the content without translation. The questions were carefully reviewed for clarity, relevance, and consistency before administration.

### 2.4 Response interpretation and cut-off values

Standardized scoring methods were used to assess participants’ knowledge and symptom profiles related to PCOS. The knowledge section consisted of 20 structured questions covering major aspects of PCOS awareness. Based on the mean score, a cut-off value of 11 was established. Participants scoring below 11 were categorized as having limited knowledge, whereas those scoring 11 or above were considered to have adequate knowledge regarding PCOS. For symptom assessment, participants were evaluated using a structured 12-item questionnaire addressing common symptoms associated with PCOS. Based on the number of reported symptoms, respondents were categorized into symptom-based risk groups. Participants reporting three or fewer symptoms were considered unlikely to have PCOS; those reporting four to eight symptoms were categorized as probable/suspected cases, while participants reporting more than eight symptoms exhibited clinical profiles highly suggestive of PCOS and were identified as requiring further clinical evaluation. These classifications were adapted from previously published epidemiological criteria (15).

### 2.5 Data collection procedures and cleaning protocols

Data collection was conducted using a systematically designed Google Form. The first section requested informed consent, offering participants a clear “Yes” or “No” option; only those who selected “Yes” were allowed to proceed, while those who declined exited the survey. Additionally, a question regarding the participant’s gender was included to exclude males from the study; if a participant selected “Male,” they were automatically redirected out of the survey. After confirming female gender, the second section gathered demographic information, including age, residence, and education details. Participants were then asked whether they had been previously diagnosed with PCOS. Those who responded “Yes” were not required to complete the symptom questionnaire and were directed to the subsequent sections of the survey. Participants who responded “No” proceeded to the third section, which consisted of a knowledge assessment with 20 targeted questions about PCOS, followed by the fourth section comprising a detailed questionnaire assessing PCOS-related symptoms and risk factors.

Before analysis, the collected data underwent a thorough cleaning process to ensure accuracy and consistency. This involved checking for and removing incomplete or duplicate responses, handling missing values, and correcting any obvious errors or inconsistencies in the dataset. Entries with missing critical information, such as consent or key demographic details, were excluded to maintain data integrity. Data formatting was standardized to allow smooth processing during statistical analysis. This careful cleaning step helped improve the quality of the data, minimizing bias and enhancing the reliability of the study results.

### 2.6 Statistical validation and psychometric analysis

The collected data were initially organized and analyzed using Microsoft Excel for descriptive statistics. However, all advanced psychometric validations and inferential statistical modeling were executed using Python (version 3.12.7). Categorical variables were summarized using frequency and percentage distributions, while continuous variables were expressed as means with standard deviations. This provided a clear overview of the demographic and clinical profiles of the participants. To ensure the reliability and dimensional accuracy of the 20-item PCOS knowledge instrument, a rigorous psychometric validation framework was applied to the binary response data. Internal consistency was evaluated using the Kuder-Richardson 20 (KR-20) formula (16) yielding a high reliability coefficient of 0.847. To evaluated individual item performance beyond classical test theory, a 2-Parameter Logistic (2-PL) item response theory (IRT) model (17) was employed to calculate the mathematical difficulty (*b*) and discrimination (*a*) parameters for each question. Furthermore, the underlying construct dimensionality was assessed via Exploratory Factor Analysis (EFA) (18). Because standard Pearson correlations attenuate relationships between dichotomous variables, a tetrachoric correlation matrix was computed prior to principal component extraction with Varimax rotation. Computations utilized the statmodels (v0.14.2), scipy (v1.11.14), numpy (v1.26.4), scikit-learn (v1.5.1) libraries. A schematic overview of the study methodology and participant selection process is given in **Figure 1**.

## 3 Result

**Figure 2:**
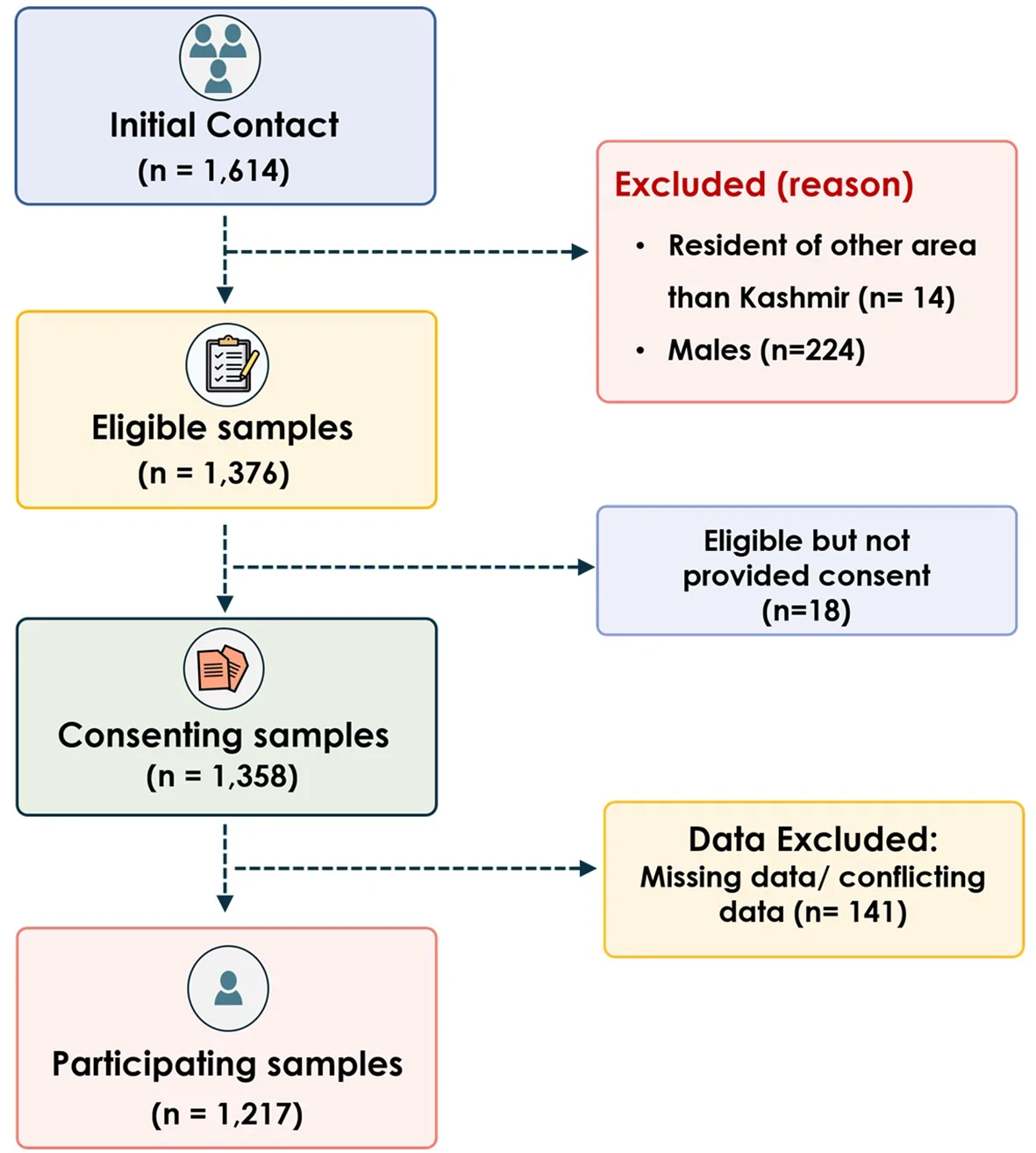
Flow diagram of participant recruitment, screening, consent and final inclusion in the analytical dataset.

Following a critical and systematic exclusion of ineligible data, a total of 1,217 participants were ultimately included in the present study (**Figure 2**), representing mean age of 21.86 ± 2.24 years (variance = 5.033). Considering the demographic features (**Table 1**), the majority of participants were from urban areas (56.61%), while 43.38% resided in rural regions. Most respondents were unmarried (98.02%), and a balanced distribution was observed across academic levels, with 34.92% in Part 2, 30.07% in Part 3, 19.06% in Part 1, and 15.94% pursuing postgraduate studies.

**Table 1:** Socio demographic distribution of study participants.

Variables	Category	Frequency (n)	Percentage (%)
Location	Rural	528/1217	43.38%
	Urban	689/1217	56.61%
Marital Status	Married	24/1217	1.97%
	Unmarried	1193/1217	98.02%
Class	Part 1	232/1217	19.06%
	Part 2	425/1217	34.92%
	Part 3	366/1217	30.07%
	PG	194/1217	15.94%

### 3.1 Clinical evaluation

**Figure 3:**
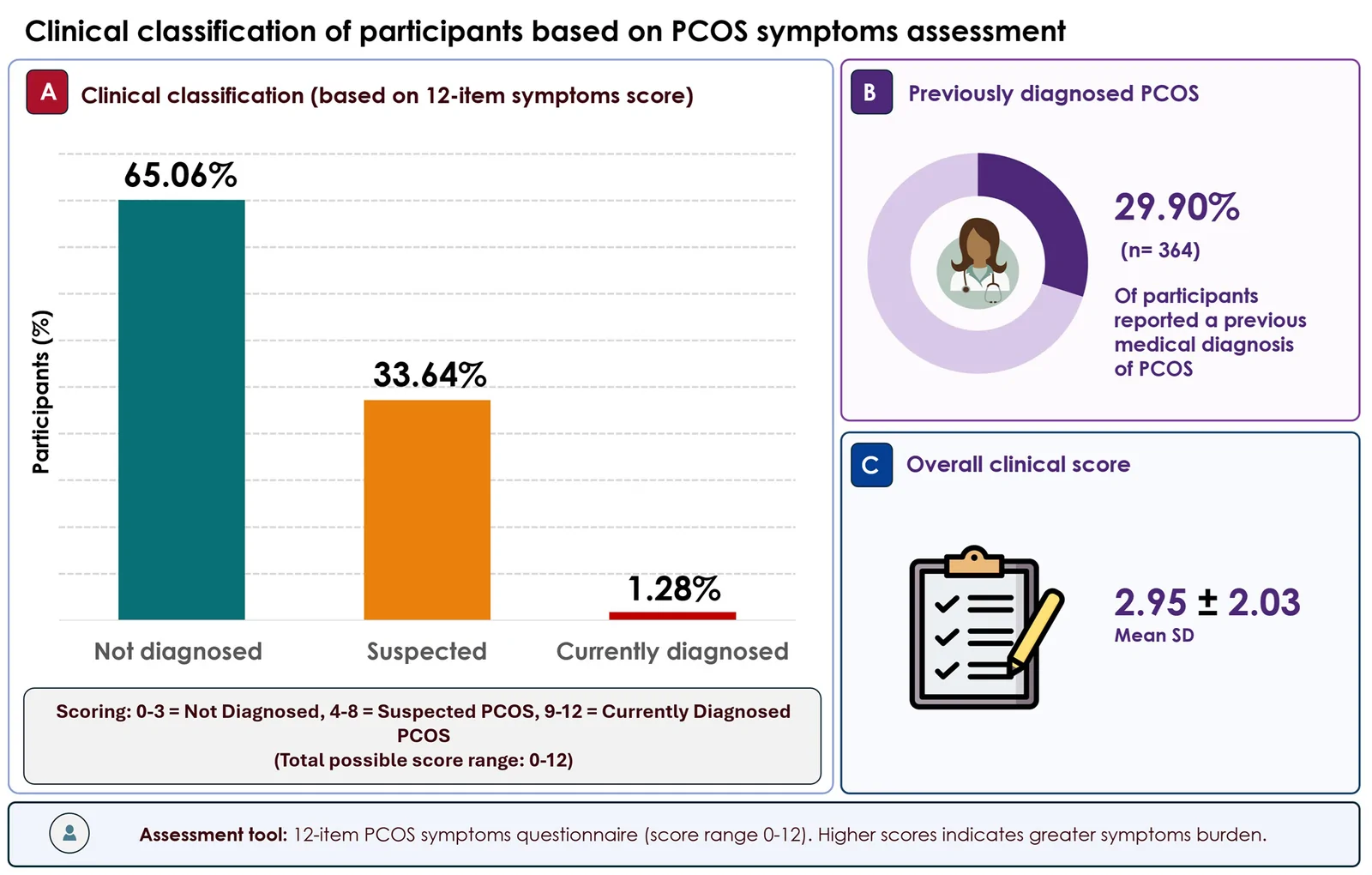
Clinical classification of participants based on PCOS symptom burden, self-reported diagnosis and overall clinical scores.

Based on the predefined symptom-based criteria (*see Section 2.4, Response Interpretation and Cut-off Values*), participants were evaluated to assess the likelihood of PCOS. The mean clinical score was 2.95 ± 2.03, indicating an overall low symptom burden within the study population. Among the 1,217 participants, 45.06% (*n* = 555) were categorized as unlikely to have PCOS (score range: 0–3), 23.58% (*n* = 287) were classified as probable/suspected cases (score range: 4–8), and 0.90% (*n* = 11) were categorized as highly suggestive of PCOS (score range: 9–12) (**Figure 3**). In addition, 29.90% (*n* = 364) of participants reported having a prior medical diagnosis of PCOS (**Table 2**), were analyzed separately and excluded from the symptom-based risk stratification.

**Table 2:** Clinical evaluation-based categorization of PCOS among study participants.

Variables	Categories (Score)	Frequency (n)	Percentage (%)
Clinical evaluation of PCOS	Not diagnosed (0-3)	555/853	65.06%
	Suspected (4-8)	287/853	33.64%
	Currently diagnosed (9-12)	11/853	1.28%
	Total (Excluding Previously Diagnosed)	853	100%
	Previously diagnosed	364/1217	29.90%
	Total	1217	100%

It should be noted that the symptom-based classification used in this study was intended solely for risk stratification and should not be interpreted as a definitive clinical diagnosis of PCOS. Overall, these findings highlight the presence of a considerable proportion of participants with symptoms suggestive of PCOS, emphasizing the importance of early screening, improved awareness, and timely clinical evaluation among young women.

### 3.2 Awareness and knowledge regarding PCOS

**Figure 4:**
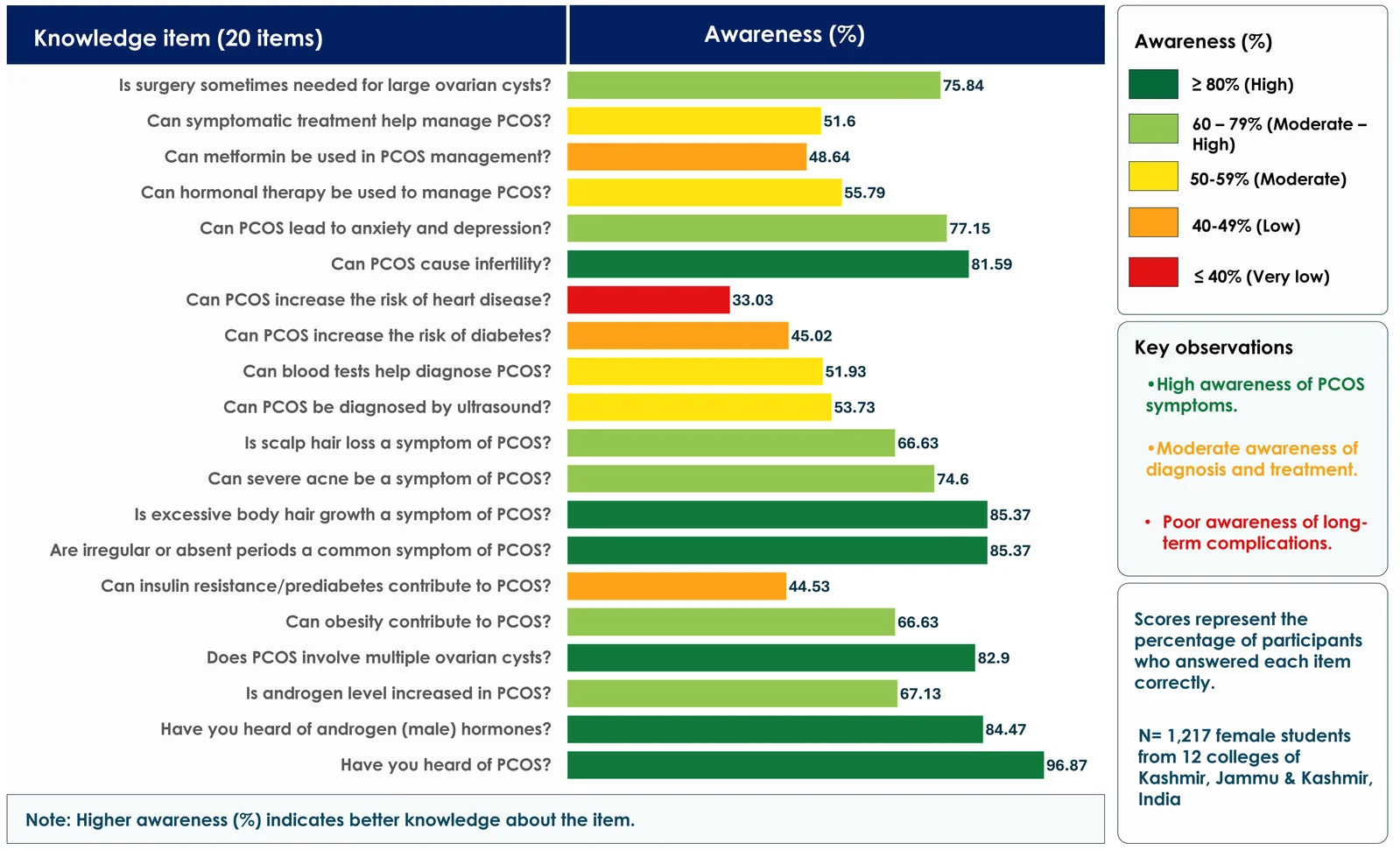
Item-level awareness of PCOS, including symptoms, risk factors, diagnosis, treatment and long-term complications, among female college students.

To assess awareness and knowledge regarding PCOS, a structured 20-item questionnaire was administered (**Table 3**). The findings revealed a high level of general awareness, with 96.8% (*n* = 1179) of respondents indicating they had heard of PCOS, and 84.47% (*n* = 1028) demonstrating familiarity with androgens such as testosterone and androstenedione (**Figure 4**). Awareness regarding the causes and risk factors of PCOS varied among participants. While 67.13% (n = 817) correctly recognized elevated androgen levels as a hallmark of PCOS, and 82.90% (n = 1009) were aware of the presence of multiple ovarian cysts, only 66.63% (*n* = 811) understood the link between obesity and PCOS. Notably, less than half, 44.53% (*n* = 542), were aware that insulin resistance or prediabetic conditions are associated with PCOS, indicating a significant gap in knowledge concerning its metabolic basis.

**Table 3:** Knowledge and awareness of PCOS among female college students.

Question type	S. no	Questions about the knowledge of PCOS	Number of responses (n=1217)	
			Yes/correct	No/incorrect
General Awareness	1	Have you heard about the term called polycystic ovary syndrome (PCOS)?	1179 (96.87%)	38
	2	Have you heard about androgen (male) hormones (e.g., testosterone and androstenedione)?	1028 (84.47%)	189
Etiological Understanding	3	In PCOS there is increased level of androgen hormone?	817 (67.13%)	400
	4	Patient suffering from PCOS have small multiple cysts in their ovaries?	1009 (82.90%)	208
	5	Does obesity cause PCOS?	811 (66.63%)	406
	6	Does a prediabetic condition (resulting from decreased insulin action in the body) contribute to the development of PCOS?	542 (44.53%)	675
Awareness of hallmark symptoms	7	Is irregular or absent menstrual cycles a common symptom of PCOS?	1039 (85.37%)	178
	8	Is excessive hair growth in various body areas like the upper lip, chin, abdomen, breasts, and thighs a symptom of PCOS?	1039 (85.37%)	178
	9	Is the presence of severe acne specifically during menstrual cycles considered a symptom indicative of polycystic ovary syndrome?	908 (74.60%)	309
	10	Is excessive hair loss from the scalp considered a symptom of PCOS?	811 (66.63%)	406
Diagnostic Awareness	11	Can PCOS be diagnosed through a vaginal ultrasound?	654 (53.73%)	563
	12	Can specific blood tests be used for diagnosing PCOS?	632 (51.93%)	585
Complications and Long-term Risks	13	Does PCOS lead to diabetes (high blood sugar)?	548 (45.02%)	669
	14	Can PCOS lead to heart diseases?	402 (33.03%)	815
	15	Does PCOS lead to infertility (inability to have children)?	993 (81.59%)	224
	16	Can PCOS lead to anxiety and depression?	939 (77.15%)	278
Awareness of Management Strategies	17	Can hormonal therapy be used to treat PCOS?	679 (55.79%)	538
	18	Can anti-diabetic medications like metformin be used to treat diabetes?	592 (48.64%)	625
	19	Can symptomatic treatment be given to relieve the symptoms of PCOS?	628 (51.60%)	535
	20	Is surgery sometimes necessary to remove large ovarian cysts?	923 (75.84%)	294

Further, recognition of PCOS symptoms was generally high among participants. A substantial majority were able to correctly identify key clinical features of PCOS, such as irregular or absent menstrual cycles (85.37%, *n* = 1039), hirsutism or excessive hair growth (85.37%, *n* = 1039), severe acne during menstrual cycles (74.6%, *n* = 908), and scalp hair loss (66.63%, *n* = 811). However, understanding of diagnostic modalities was comparatively moderate. Only 53.73% (*n* = 654) of participants recognized that vaginal ultrasound could be used as a diagnostic tool, and 51.93% (*n* = 632) were aware that specific blood tests are also employed in diagnosing PCOS. When examining knowledge of potential complications and long-term health risks, awareness again varied. Approximately 45.02% (*n* = 548) understood that PCOS could lead to diabetes, while only 33% (*n* = 402) associated it with cardiovascular disease. On the other hand, there was strong recognition of reproductive and psychological complications, with 81.59% (*n* = 993) acknowledging infertility as a possible outcome and 77.15% (*n* = 939) recognizing an association with anxiety and depression. Regarding the awareness of management strategies, about half of the individuals are aware that hormonal therapy (55.79%) and anti-diabetic medications like metformin (48.64%) can be used to manage PCOS and diabetes, respectively. Additionally, a majority (75.84%) understand that surgery may be necessary to remove large ovarian cysts. Overall, the findings indicate good awareness of the gynecological manifestations of PCOS among participants, but comparatively limited understanding of its metabolic complications, diagnostic methods, and associated long-term health risks.

## 4 Statistical analysis and reliability validation

The psychometric properties of the PCOS knowledge instrument were evaluated through a combination of reliability and validity analyses. Internal consistency reliability was assessed using the Kuder-Richardson formula 20 (KR-20), while Item Response Theory (IRT) based on the two-parameter logistic (2-PL) model was employed to examine item difficulty and discrimination characteristics. Furthermore, Exploratory Factor Analysis (EFA) was conducted to investigate the latent factor structure underlying the questionnaire. Collectively, these analyses provided a comprehensive assessment of the instrument’s measurement performance and construct validity.

### 4.1 Kuder-Richardson formula 20 (KR-20)

Internal consistency reliability of the 20-item PCOS knowledge instrument was evaluated using the Kuder-Richardson Formula (KR-20) (16). This methodology was selected as the optimal statistical measure because the instrument utilizes a dichotomous scoring matrix (1=Yes, 0=No), making standard Cronbach’s Alpha mathematically inappropriate (19, 20). The KR-20 reliability coefficient was derived using the following equation:

(2) $$KR_{20}=\frac{k}{k-1}(1-\frac{\sum pq}{{\text{σ}}_{X}^{2}})$$

where *k* represents the total number for test items, *p* represents the proportion of positive/correct responses for each individual item, *q* represents the proportion of negative/incorrect responses for each item, *∑pq* represents individual item variance, and σ^2x^ represents the total variance. The resulting KR-20 coefficient of 0.847 significantly exceeds the widely accepted psychometric threshold of 0.70 for exploratory research (21). The high degree of internal consistency confirms that the 20 items reliably intercorrelate and uniformly measure a single underlying construct of PCOS awareness across the sampled demographic. Detailed item-level KR-20 reliability statistics are reported in **Supplementary Table 1**.

### 4.2 Item response theory analysis

To evaluate the psychometric properties of the 20-item PCOS knowledge instrument beyond classical test theory, an Item Response Theory (IRT) framework was applied (22). Specifically, a 2-parameter logistic (2-PL) model was utilized to accommodate the dichotomous nature of response data. Unlike classical models that assume equal weighting across all equations, the 2-PL model mathematically isolates two independent parameters for each item: item difficulty (*b*) and item discrimination (*a*) (23, 24). The item difficulty (*b)* represents the threshold location on the latent trait continuum ( θ) where a participant has a 50% probability of answering correctly. Higher positive values indicate higher difficulty. The item discrimination (*a*) is the slope of the item characteristic curve (ICC) at the difficulty threshold, representing how effectively the item differentiates between participants with varying levels of underlying PCOS knowledge. The probability of a correct response to item *j* by participant *i* was modeled using the following mathematical equation:

(3) $$P(X_{ij}=1|\theta_{i})=\frac{\exp(a_{j}(\theta_{i}-b_{j}))}{1+\exp(a_{j}(\theta_{i}-b_{j}))}$$

where *P* is the probability that participant *i* answers question *j* correctly.

The IRT analysis revealed distinct variations in both difficulty and discrimination across the tested domains, demonstrating that the instrument maps effectively across the latent trait continuum. Regarding item difficulty, the most challenging items (*b>0*) were strictly associated with the long-term metabolic and systemic complications of PCOS, specifically the risks of heart disease (*a* = 2.163, *b* = 0.631), prediabetic mechanisms (*a* = 1.669, *b* = 0.255), and the utility of anti-diabetic medications (*a* = 1.675, *b* = 0.106). Conversely, the basic recognition of the syndrome’s nomenclature (“Have you heard about the term PCOS?”) served as the easiest item (*a* = 1.433, *b* = -3.107), establishing a foundational knowledge baseline.

Concurrently, the discrimination parameters (*a*) generally exceeded 1.0, signifying robust psychometric utility across the scale. The item addressing excessive hair growth exhibited the highest discrimination slope (*a* = 2.386, *b* = -1.349), suggesting that a failure to recognize this primary physical manifestation is highly predictive of an overall deficit in construct knowledge. In contrast, the item regarding vaginal ultrasound diagnosis exhibited the weakest discrimination (*a* = 0.899, *b* = -0.170), indicating that responses concerning this specific clinical methodology provided the least statistical value in differentiating between low-knowledge and high-knowledge participants.

**Figure 5:**
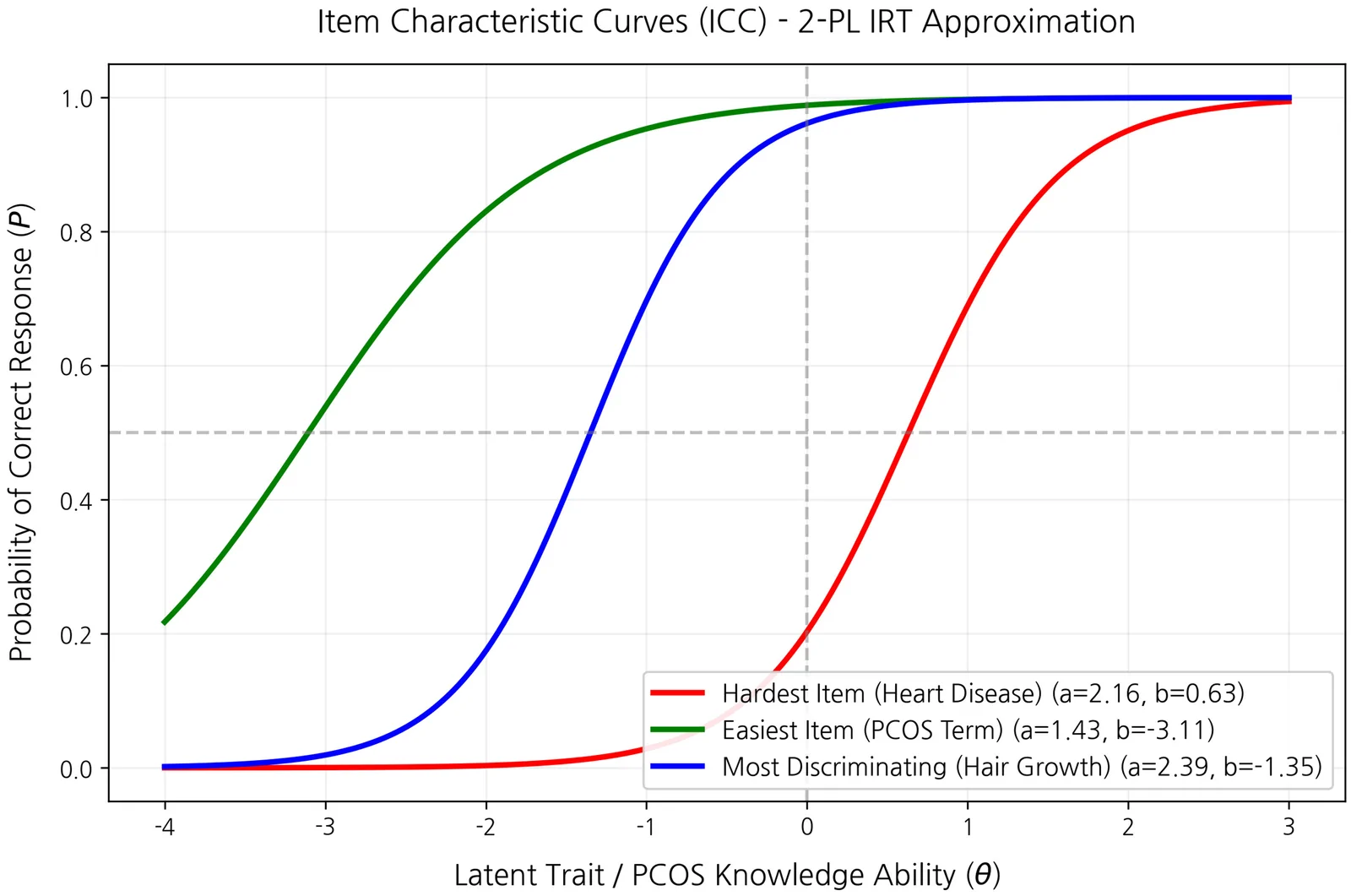
Item characteristic curves (ICCs) from the 2-Parameter Logistic (2-PL) item response theory model illustrating the relationship between latent PCOS knowledge (*θ*) and the probability of a correct response.

**Figure 5** presents the Item Characteristic Curves (ICCs) derived from the 2-PL IRT model. As shown in **Figure 5**, the green curve represents the easiest item (“Have you heard about the term PCOS?”), the red curve represents the most difficult item (“Can PCOS lead to heart diseases?”), and the blue curve represents the most discriminating item (“Is excessive hair growth a symptom of PCOS?”). The horizontal position of the curve reflects item difficulty, whereas the steepness of the curve indicates its ability to discriminate between participants with different levels of PCOS knowledge. The individual IRT values are reported in **Supplementary Table 2**.

### 4.3 Exploratory Factor Analysis (EFA)

To investigate the underlying dimensionality of the 20-item PCOS knowledge instrument, an Exploratory Factor Analysis (EFA) (19) was conducted. Because the item-level date was dichotomous, standard Pearson product-moment correlations were deemed mathematically inappropriate, as they inherently attenuate the magnitude of relationships between binary variables. Instead, a tetrachoric correlation matrix was computed to serve as the foundation for the EFA (25). The tetrachoric correlation (*r_tet_*) estimates the Pearson correlation that would be obtained if the two binary measured variables were continuous and normally distributed. For any two dichotomous items (*X* and *Y*) forming a 2×2 contingency table with cell frequencies *a, b, c*, and *d*, the approximation of the tetrachoric correlation was calculated using the cosine function:

(4) $$r_{tet}\approx Cos(\frac{\pi }{1+\sqrt{\frac{a.d}{b.c}}})$$

Prior to factor extraction, the suitability of the data for structure detection was evaluated. The Kaiser-Meyer-Olkin (KMO) measure of sampling adequacy was 0.928, indicating excellent factorability, and Bartlett’s Test of Sphericity was highly significant $(\chi^{2}\;(190)\;=\;6985.97,\;p<0.001),\;$statistically justifying the use of factor analysis. Following the generation of the tetrachoric correlation matrix, principal component extraction was performed. The number of retained factors was determined using the Kaiser Criterion, which strictly extracts factors with eigenvalues greater than 1.0 (*λ >* 1). To maximize the interpretability of the extracted dimensions and achieve a simple structure, an orthogonal Varimax rotation was applied (26).

The EFA yielded a distinct three-factor solution that demonstrated excellent model fit, cumulatively explaining 69.10% of the total variance in the latent construct, mathematically classifying PCOS knowledge into three discrete cognitive domains (detailed statistical values are provided in **Supplementary Table 3**). Factor loadings exceeding an absolute threshold of 0.30 were considered significant for item inclusion. This dimensional clustering revealed a clear structure regarding how PCOS knowledge is acquired and conceptualized among the sample demographic. The first factor, defined as Basic Symptoms and Primary Manifestation, captures foundational, surface-level knowledge encompassing direct physical and reproductive consequences, such as hirsutism, irregular cycles, ovarian cysts, and infertility. The second factor, Systemic and Metabolic Complications, independently isolates deeper endocrinological understanding by exclusively grouping items concerning long-term, non-reproductive pathologies, specifically the cardiovascular and glycemic risks associated with the syndrome. Finally, a tertiary factor, categorized as Clinical Interventions, captures awareness regarding the pharmacological and symptomatic management of the condition.

## 5 Discussion

PCOS is a common endocrine disorder affecting women of reproductive age and is associated with reproductive, metabolic, and psychological complications. Reported global prevalence varies widely from 5% to 26%, largely depending on diagnostic criteria and population characteristics (27, 28). Against this global background, the present study was conducted to assess the burden and risk profile of PCOS among college-aged women in Kashmir. The study included 1,217 participants with a mean age of 21.86 ± 2.24 years. Approximately 29.90% of participants reported a prior medical diagnosis of PCOS, while 23.58% were categorized as probable/suspected cases based on symptom-based risk assessment. In addition, 0.90% of participants were classified as highly suggestive of PCOS according to predefined symptom-based criteria. Depending on the study population and diagnostic criteria employed, similar studies from various regions have also reported a high prevalence of PCOS among young women and college-going populations, with prevalence estimates ranging from 3.7% to 36% (29–33). In Kashmir, reported prevalence rates range from 29% to 35%, although these estimates vary according to demographic characteristics and diagnostic criteria (12).

To better interpret these findings, the sociodemographic profile of the study population was also analyzed. The sociodemographic profile of the participants demonstrated that the majority were from urban areas (56.61%), while 43.38% belonged to rural regions of the Kashmir valley. Previous studies have suggested a potential link between urban environments and PCOS among urban women with sedentary lifestyles (34), unhealthy dietary patterns (35–38), obesity, and insulin resistance (39). In the present study, these factors were not evaluated and therefore no such associations can be inferred. Nearly all participants were unmarried (98.02%) college students, with representation across different academic levels (**Table 1**). These findings reflect a predominantly young, urban-based student population. While we did not statistically test for demographic associations, these characteristics serve as a descriptive backdrop for observing overall PCOS awareness and symptom patterns among female university students in Kashmir.

In addition to demographic characteristics, the study also assessed awareness and knowledge regarding PCOS, with 96.8% of participants reporting that they had heard of the disorder. This high level of awareness may reflect increasing exposure to PCOS-related information through educational and media platforms (40–43). However, despite good general awareness, notable gaps remained regarding the underlying causes, metabolic basis, and long-term complications of PCOS (**Table 3**). Similar findings have been reported in previous studies, which also identified insufficient awareness and understanding of PCOS among females (44–46). To further evaluate the depth of this awareness, participants’ understanding of hormonal and diagnostic aspects of PCOS was also assessed. Improving awareness among college-going women is important for promoting early diagnosis and reducing the risk of reproductive, metabolic, and psychological complications associated with PCOS (47). The study further showed that 84.47% of participants were familiar with androgens such as testosterone and androstenedione, indicating a general awareness that PCOS is associated with hormonal imbalance. A considerable proportion also recognized hyperandrogenism and polycystic ovarian morphology (PCOM) as important features of PCOS. However, understanding of the relationship between these diagnostic features remained limited. Since hyperandrogenism is a key diagnostic criterion of PCOS (27, 48), inadequate awareness of its clinical significance may contribute to delayed recognition and diagnosis of the disorder.

A notable gap was observed in awareness regarding the association between obesity and PCOS, with only 66.63% of participants recognizing this link (49, 50). This is concerning because obesity can worsen PCOS symptoms (51) and increase the risk of metabolic complications such as type 2 diabetes, while PCOS itself may contribute to weight gain. Previous studies have similarly reported a strong association between obesity and PCOS, highlighting the importance of awareness for effective prevention and management (52). The most significant knowledge gap identified in the study was related to the metabolic basis of PCOS, as only 44.53% of participants recognized insulin resistance or prediabetic conditions as contributing factors to PCOS. This is concerning because insulin resistance is considered a major underlying mechanism in PCOS pathogenesis and is closely associated with hyperandrogenism, ovulatory dysfunction, and long-term metabolic complications (53). Limited awareness of this relationship may contribute to delayed diagnosis and reduced understanding of the importance of lifestyle modifications. Furthermore, awareness of metabolic complications was comparatively low, with only 45.02% of participants recognizing the association of PCOS with diabetes, and 33% identifying its link with cardiovascular disease. These findings highlight an important gap in understanding the long-term metabolic consequences of PCOS (54).

Furthermore, the findings demonstrated relatively good awareness of common clinical manifestations of PCOS, including irregular menstrual cycles, hirsutism, acne, and scalp hair loss, suggesting familiarity with its more visible symptoms that may prompt early medical consultation (15). In comparison, awareness of long-term complications showed variability among participants. A large proportion recognized infertility (81.59%) and the association of PCOS with psychological conditions such as anxiety and depression (77.15%). This aligns with previous studies reporting higher rates of anxiety and depressive symptoms among women with PCOS compared to healthy individuals (55, 56). However, knowledge regarding diagnostic methods was comparatively limited, with only about half of the participants identifying ultrasonography and hormonal blood tests as key diagnostic tools. This gap in understanding may contribute to delayed diagnosis or underdiagnosis even when symptoms are recognized. Therefore, improving awareness of evidence-based diagnostic approaches is essential for promoting timely clinical evaluation and appropriate management of PCOS (57). Similarly, awareness regarding management strategies was moderate, with nearly half of the participants recognizing hormonal therapy and metformin as treatment options. Nevertheless, a substantial proportion (75.84%) incorrectly believed that surgery is commonly required to remove ovarian “cysts,” reflecting a persistent misconception. In reality, these structures are typically immature follicles rather than true cysts requiring surgical intervention (58).

Overall, the findings show good recognition of visible symptoms of PCOS, but limited awareness of its metabolic, psychological, diagnostic, and management aspects. This highlights key knowledge gaps among college-going women. Targeted health education in schools and universities is needed to improve understanding of metabolic risks, diagnostic methods, and evidence-based treatments, while correcting misconceptions. Such efforts may support earlier diagnosis and improved long-term health outcomes by reducing metabolic and psychological complications.

To objectively quantify and map this heterogeneous understanding, our study employed a rigorous psychometric and statistical framework, revealing critical insights into how PCOS knowledge is structured among young women. A 2-Parameter Logistic (2-PL) Item Response Theory (IRT) model was utilized to calculate the mathematical difficulty and discrimination of each question. The IRT analysis confirmed that the most poorly understood aspects of PCOS are exclusively linked to its long-term metabolic complications. The four most difficult items on the latent trait continuum, the links to heart disease, prediabetic conditions, diabetes, and the use of anti-diabetic medications, all exhibited high mathematical difficulty thresholds (*b >* 0.10). Conversely, questions regarding superficial symptoms, such as excessive hair growth, demonstrated the highest item discrimination (*a* = 2.386), indicating that a failure to recognize basic physical manifestations is highly predictive of overall low health literacy regarding the syndrome.

Furthermore, Exploratory Factor Analysis (EFA) via tetrachoric correlation mathematically validated that PCOS knowledge is not acquired as a single unified concept, but is rigidly compartmentalized into three distinct cognitive domains: Basic Symptoms, Systemic & Metabolic Complications, and Clinical Interventions. This dimensional clustering explains why a participant can easily recognize irregular cycles and acne (Factor 1) while remaining entirely unaware of the severe cardiovascular and glycemic risk (Factor 2).

## 6 Strengths, limitations, and future perspectives

The present study has several strengths, including a large sample size (*n* = 1,217), which enhances statistical power and improves the robustness of findings. The use of a structured questionnaire adapted from established studies ensured comprehensive assessment of both symptom profile and awareness levels. Inclusion of participants from both urban and rural areas further improved sample diversity, while strict ethical procedures and data cleaning strengthened data quality and reliability.

However, several limitations should be acknowledged. The use of convenience sampling and voluntary online recruitment may have introduced selection bias, while the total number of students invited and the response rate were not prospectively recorded, potentially limiting the generalizability of the findings. The cross-sectional design precludes causal inference, and reliance on self-reported data may have introduced recall and reporting bias. Furthermore, the absence of clinical and biochemical confirmation, anthropometric measurements (e.g., BMI), detailed menstrual and family history data, together with the use of an English-only questionnaire, may have limited the comprehensive assessment of PCOS risk and participant inclusivity. Finally, no multivariable analyses were performed, as the study was designed primarily for descriptive assessment of PCOS burden and awareness. Future studies should employ population-based sampling, longitudinal study designs, and clinical and biochemical assessments to provide more robust estimates of PCOS burden. The use of culturally adapted questionnaires and the implementation of targeted educational and screening programs in educational institutions may further improve early identification and management of PCOS.

## 7 Conclusion

This study highlights a substantial burden of previously undiagnosed, symptom-screened PCOS risk among young women in Kashmir, together with important gaps in awareness, particularly regarding its metabolic consequences and diagnostic approaches. These findings underscore the need for targeted health education, awareness campaigns, and screening programs within educational institutions to promote early identification and timely clinical evaluation. Policymakers and healthcare providers should integrate PCOS awareness and preventive strategies into student health programs to improve early recognition, facilitate appropriate management, and reduce the long-term reproductive, metabolic, and psychological burden of PCOS.

## Data Availability

The raw data supporting the conclusions of this article will be made available by the authors, without undue reservation.
