# Supplementary material for: Prevalence of polycystic ovary syndrome among college students in kashmir (J&K) india: a cross-sectional study
Source: Front Endocrinol (Lausanne). 2026 Aug 26;17:1912986. doi: 10.3389/fendo.2026.1912986 (PMC13553269; doi:10.3389/fendo.2026.1912986)
Supplement: Supplementary file 1 [file DataSheet1.pdf]

Supplementary Material for: Prevalence of Polycystic Ovary Syndrome among college students in Kashmir (J&K) India: A Cross-Sectional Study

SUPPLEMENTARY DATA

Table S1. Item difficulty and variance statistics for the PCOS knowledge instrument

Item	Question	Difficulty (p)	Variance (pq)
1	Have you heard about the term called polycystic ovary syndrome (PCOS)?	0.9688	0.0302
2	Have you heard about androgen (male) hormone (e.g., testosterone and androstenedione)?	0.8447	0.1312
3	In PCOS there is increased level of androgen hormone?	0.6713	0.2206
4	Patients suffering from PCOS have small multiple cysts in their ovaries?	0.8291	0.1417
5	Does obesity cause PCOS?	0.6664	0.2223
6	Does a prediabetic condition (resulting from decreased insulin action in the body) contribute to the development of PCOS?	0.4454	0.2470
7	Is irregular or absent menstrual cycles a common symptom of PCOS?	0.8537	0.1249
8	Is excessive hair growth in various body areas like the upper lip, chin, abdomen, breasts, and thighs a symptom of PCOS?	0.8537	0.1249
9	Is the presence of severe acne specifically during menstrual cycles considered a symptom indicative of PCOS?	0.7461	0.1894
10	Is excessive hair loss from the scalp considered a symptom of PCOS?	0.6664	0.2223
11	Can PCOS be diagnosed through a vaginal ultrasound?	0.5374	0.2486
12	Can specific blood tests be used for diagnosing PCOS?	0.5193	0.2496
13	Does PCOS lead to diabetes (high blood sugar)?	0.4503	0.2475
14	Can PCOS lead to heart diseases?	0.3303	0.2212
15	Does PCOS lead to infertility (inability to have children)?	0.8159	0.1502
16	Can PCOS lead to anxiety and depression?	0.7716	0.1763
17	Can hormonal therapy be used to treat PCOS?	0.5579	0.2466
18	Can anti-diabetic medications like metformin be used to treat diabetes?	0.4864	0.2498
19	Can symptomatic treatment be given to relieve the symptoms of PCOS?	0.5604	0.2464
20	Is surgery sometimes necessary to remove large ovarian cysts?	0.7584	0.1832

Table S2. Item Response Theory (2-PL) Parameter Estimates for the PCOS knowledge instrument

Item	Question	Discrimination (a)	Difficulty (b)
1	Can PCOS lead to heart diseases?	2.163	0.631
2	Does a prediabetic condition (resulting from decreased insulin action in the body) contribute to the development of PCOS?	1.669	0.255
3	Does PCOS lead to diabetes (high blood sugar)?	1.781	0.235
4	Can anti-diabetic medications like metformin be used to treat diabetes?	1.675	0.106
5	Can specific blood tests be used for diagnosing PCOS?	1.203	-0.044
6	Can hormonal therapy be used to treat PCOS?	1.810	-0.145
7	Can symptomatic treatment be given to relieve the symptoms of PCOS?	1.640	-0.168
8	Can PCOS be diagnosed through a vaginal ultrasound?	0.899	-0.170
9	Does obesity cause PCOS?	1.755	-0.565
10	Is excessive hair loss from the scalp considered a symptom of PCOS?	1.590	-0.597
11	In PCOS there is increased level of androgen hormone?	1.577	-0.621
12	Is the presence of severe acne specifically during menstrual cycles considered a symptom indicative of polycystic ovary syndrome?	1.754	-0.913
13	Can PCOS lead to anxiety and depression?	2.153	-0.951
14	Is surgery sometimes necessary to remove large ovarian cysts?	1.270	-1.162
15	Does PCOS lead to infertility (inability to have children)?	2.051	-1.196
16	Patients suffering from PCOS have small multiple cysts in their ovaries?	2.213	-1.235
17	Is excessive hair growth in various body areas like the upper lip, chin, abdomen, breasts, and thighs a symptom of PCOS?	2.386	-1.349
18	Have you heard about androgen (male) hormone (e.g., testosterone and androstenedione)?	2.094	-1.353
19	Are irregular or absent menstrual cycles a common symptom of PCOS?	1.936	-1.453
20	Have you heard about the term called polycystic ovary syndrome (PCOS)?	1.433	-3.107

Table S3. Exploratory Factor Analysis (EFA) factor loadings for the PCOS knowledge instrument

S.No.	Questionnaire Item	Factor 1 (Symptoms)	Factor 2 (Complications)	Factor 3 (Treatment)
1	Is excessive hair growth in various body areas like the upper lip, chin, abdomen, breasts, and thighs a symptom of PCOS?	0.724	–	–
2	Is irregular or absent menstrual cycles a common symptom of PCOS?	0.710	–	–
3	Patients suffering from PCOS have small multiple cysts in their ovaries?	0.700	–	–
4	Have you heard about androgen (male) hormone (e.g., testosterone and androstenedione)?	0.687	–	–
5	Does PCOS lead to infertility (inability to have children)?	0.626	–	–
6	Can PCOS lead to anxiety and depression?	0.573	–	–
7	Is the presence of severe acne specifically during menstrual cycles considered a symptom indicative of polycystic ovary syndrome?	0.519	–	–
8	Does obesity cause PCOS?	0.412	–	–
9	Does PCOS lead to diabetes (high blood sugar)?	–	0.737	–
10	Can PCOS lead to heart diseases?	–	0.702	–
11	Does a prediabetic condition (resulting from decreased insulin action in the body) contribute to the development of PCOS?	–	0.557	–
12	Can anti-diabetic medications like metformin be used to treat diabetes?	–	0.500	–
13	Can specific blood tests be used for diagnosing PCOS?	–	0.372	–
14	In PCOS there is increased level of androgen hormone?	–	0.348	–
15	Can hormonal therapy be used to treat PCOS?	–	–	0.440
16	Can symptomatic treatment be given to relieve the symptoms of PCOS?	–	–	0.413
